# A 40-Day Journey to Better Health: Utilizing the DanielFast to Improve Health Outcomes in Urban Church-Based Settings

**DOI:** 10.3390/healthcare6010025

**Published:** 2018-03-05

**Authors:** Nicole A. Vaughn, Darryl Brown, Beatriz O. Reyes, Crystal Wyatt, Kimberly T. Arnold, Elizabeth Dalianis, Paula J. Kalksma, Caryn Roth, Jason Langheier, Maria Pajil-Battle, Meg Grant

**Affiliations:** 1 Department of Health & Exercise Sciences, School of Health Professions, Rowan University, Glassboro, NJ 08028, USA; kalksma@rowan.edu; 2 Department of Biomedical Sciences, Cooper Medical School of Rowan University, Camden, NJ 08103, USA; 3 Department of Family Medicine, School of Osteopathic Medicine, Rowan University, Stratford, NJ 08084, USA; 4 Department of Health Management & Policy, School of Public Health, Drexel University, Philadelphia, PA 19104, USA; drb48@drexel.edu; 5 Department of Anthropology, Global Health Studies Program, Northwestern University, Evanston, IL 60208, USA; beatriz.reyes@northwestern.edu; 6 Ride & Rebuild, LLC, Philadelphia, PA 19151, USA; cwyatt1119@gmail.com; 7 Department of Health Policy & Management, Bloomberg School of Public Health, Johns Hopkins University, Baltimore, MD 21205, USA; karnol14@jhmi.edu; 8 Community College of Philadelphia, Philadelphia, PA 19130, USA; edalianis@ccp.edu; 9 Zipongo, Inc., San Francisco, CA 94133, USA; carynroth@gmail.com (C.R.); jason.langheier@zipongo.com (J.L.); 10 AmeriHealth Caritas Partnership, Philadelphia, PA 19113, USA; riarudybattle@aol.com; 11 Keystone First, Philadelphia, PA 19113, USA; mgrant@keystonefirstpa.com

**Keywords:** African American, community intervention, urban settings, health disparity, technology, weight loss, nutrition education

## Abstract

*Background:* As the costs associated with obesity increase, it is vital to evaluate the effectiveness of chronic disease prevention among underserved groups, particularly in urban settings. This research study evaluated Philadelphia area Keystone First members and church participants enrolled in a group health education program to determine the impact of the Daniel Fast on physical health and the adoption of healthy behaviors. *Methods:* Participants attended six-weekly health education sessions in two participating churches, and were provided with a digital healthy eating platform. *Results:* There was a statistically significant decrease from baseline to post assessment for weight, waist circumference and cholesterol. Participants reported a significant improvement in their overall well-being, social and physical functioning, vitality and mental health. *Conclusion:* Results of this study demonstrate that dietary recommendations and comprehensive group health education delivered in churches and reinforced on a digital platform can improve physical health, knowledge and psychosocial outcomes.

## 1. Introduction

Seven of the top 10 leading causes of death in the U.S. are due to chronic diseases. 8.6% of the nation’s $2.7 trillion annual health care expenditure goes toward treating these diseases. Approximately 50% of adults (117 million people) have one or more chronic diseases or conditions [1]. Cardiovascular disease (CVD), diabetes and obesity are the most prevalent and costly chronic diseases. The top two leading causes of death, heart disease and cancer, account for 46% of deaths in the U.S. and over 80% of all health care costs [1,2]. Cardiovascular disease claims the lives of approximately 600,000 Americans each year and CVD and stroke, combined, cost an estimated $316.1 billion [3,4]. In 2010, more than one-third of adults were obese [3]. Medical costs related to obesity were estimated to exceed $147 billion. Additionally, diabetes expenditures approached $245 billion and affect over 29 million people in the U.S. [5,6]. 

Despite the complex etiologies of many chronic diseases, many are preventable through healthy behaviors, such as diet and regular physical activity. Studies have shown that individuals who lose weight, modify their eating behaviors, and increase their physical activity can prevent many chronic conditions (e.g., diabetes, hypertension, and cardiovascular disease) [7]. As the physical, economic and societal cost associated with overweight and obesity increase, it is vital to evaluate the effectiveness of chronic disease prevention interventions among high-risk groups, especially African Americans living in urban settings. 

Health disparities do exist for some racial and ethnic groups with particularly large health disparities and inequities among African Americans [8]. Inadequate access to health care, lack of education, poverty, racism, and community conditions lead to health disparities, resulting in poor health outcomes and higher healthcare costs [8]. Among African Americans, one out of every two persons is obese, which increases the risk for developing chronic conditions. According to the Centers for Disease Control and Prevention (CDC), African Americans aged 18–49 years are twice as likely to die from heart disease compared to Caucasians, and African Americans aged 35–64 years are 50% more likely to have high blood pressure than Caucasians [9,10].

Currently, 31.8% of Pennsylvanians are obese. Of this third, approximately 36.4% are African Americans [9]. Among the top 10 most populous cities, Philadelphia experiences the highest prevalence of hypertension (34.5%), type II diabetes (16%), and adult obesity (32%), exceeding the national average by 3.7%, 3.3%, and 4.5% respectively [11]. Stark racial/ethnic health disparities exist for all of these diseases, with African-Americans carrying the heaviest burden of hypertension (47.1%), type II diabetes (20.2%), and obesity (41.8%) compared to non-Hispanic Whites, Hispanic/Latinos, and Asians [11]. In order to reduce adverse health outcomes associated with obesity, especially for African Americans, it is important to design and implement targeted programs to improve health behaviors where people live. 

### Background

The “40-Day Journey to Better Health (40-Day Journey)” program, designed by AmeriHealth Caritas Partnership, encourages individuals to change their nutrition and physical activity behaviors during the Lenten season (a Christian religious observance that occurs 40 days before Easter and promotes fasting and sacrifice). The program included a group health education curriculum, digital engagement, and the Daniel Fast (Fast), a plant-based diet based on the book of Daniel in the Bible. The Fast promotes eating fruits, vegetables and whole foods, drinking water, and eliminating sweeteners and bread for 40 days [12]. The Fast excludes the consumption of meat, dairy, sweeteners, and bread, consistent with a strict vegan diet [12,13]. AmeriHealth Caritas Partnership and Keystone First worked with Zipongo, a web- and app-based nutrition platform, and a local Philadelphia chef to develop a series of recipes and meal plans based on the diet. Zipongo introduced a digital healthy eating platform to the intervention that included sending recipes, meal plans and text messages to support users in adopting and maintaining the Fast. Also, Healthy Measures, Inc. was contracted to collect biometric data and the university-based research team collected behavioral health data and evaluated the study.

Recently, the Daniel Fast has gained interest and popularity among congregations across the U.S. Many church leaders have presented the plan to their congregants despite a paucity of research on its impact on health and the programming necessary to drive adoption. Although there is limited research, the Fast has been promoted by prominent and influential figures (e.g., Pastor Rick Warren) and some members of the medical community.

Despite the limited research, prior study results have suggested a positive impact on health outcomes. Specifically, Bloomer et al. (2010) found that people who adopted the Fast experienced improvements in many metabolic and cardiovascular disease risk factors and concluded that the Fast is an effective strategy in chronic disease prevention [14]. Compared to baseline, white blood cell count, blood urea nitrogen, protein, total cholesterol, low-density lipoprotein (LDL) cholesterol, high-density lipoprotein (HDL) cholesterol, and blood pressure all significantly lowered after the Fast. Although not statistically significant, there were also clinically meaningful outcomes which included improvements in insulin, homeostatic model assessment of insulin resistance (HOMA-IR), and C-Reactive Protein (CRP) [14]. In another evaluation of the 21-day version of the Fast, participants had improvements in selected biomarkers of antioxidant status and oxidative stress [13]. Furthermore, individuals who had adopted either a traditional or a modified Daniel Fast diet had significant improvements in blood lipids and reduced inflammation [15,16]. A 2010 review concluded that the Daniel Fast can yield improved health outcomes of reduced blood pressure, blood lipids, insulin sensitivity, and oxidative stress [17]. If the traditional or modified diet is sustained over time, individuals may improve blood pressure and blood lipids [14,15,16]. 

In addition to diet change, previous randomized control studies have shown similar successes changing health behaviors through text messaging in order to increase support [18]. A weight loss program targeted at obese African American women found that the group receiving daily text messages lost weight while the control group receiving only didactic health education gained weight [19]. The researchers hypothesized that the text messaging helped with daily self-monitoring of diet, which has previously been linked to increased weight loss. Another randomized controlled trial which compared weight loss for participants receiving paper-based health education to those also receiving two-five daily text messages found that the text messaging group lost significantly more weight [20].

The Zipongo digital healthy eating platform was used in this intervention to support participants adopting new healthy eating behaviors. During the maintenance phase (six-week–six-month follow-up), in accordance with the Fast, participants were provided specific recipes and meal plans in print, web, smartphone and text messaging format. Text messaging included dinner recipe recommendations and weekly grocery lists with discounts from each user’s preferred grocery stores. Prior programs have assessed text messaging to help people with diabetes improve habits such as fruit and vegetable consumption with some success, but a comprehensive healthy eating program, or one using the Fast with churches, has not been previously explored. 

The purpose of this research study was to evaluate the impact of the 40-Day Journey—a faith-based dietary guidance program paired with digital and text messaging platform—on weight change, health knowledge, health behaviors and clinical outcomes in churches located in an urban setting. Participants were advised to adopt the Daniel Fast for their 2013 observation of Lent. 

## 2. Materials and Methods 

### 2.1. Design

Participants attended a six-week intervention to improve eating and exercising behaviors at two predominantly African American churches in Philadelphia, PA, USA. These churches served as the site for the 40-Day Journey. The active phase of this intervention started in February 2013, follow-up occurred at six months, and data analysis occurred in 2014. The Drexel University Institutional Review Board approved this study protocol. 

Two predominantly African American churches were selected by AmeriHealth based on established pastoral relationships, interest in health programming, congregation size, space availability and being located in areas with high chronic disease statistics (i.e., overweight, obesity, CVD, etc.). 

Participants were then recruited by AmeriHealth Caritas Partnership through flyers. Keystone First identified health plan members with type 2 diabetes in the zip codes surrounding the two churches. They were also provided transportation to the weekly sessions if needed. All participants gave informed consent prior to enrollment. All participants were eligible to participate in weekly raffle drawings as an incentive (e.g., blender, pedometers, cookbooks).

**Figure 1:**
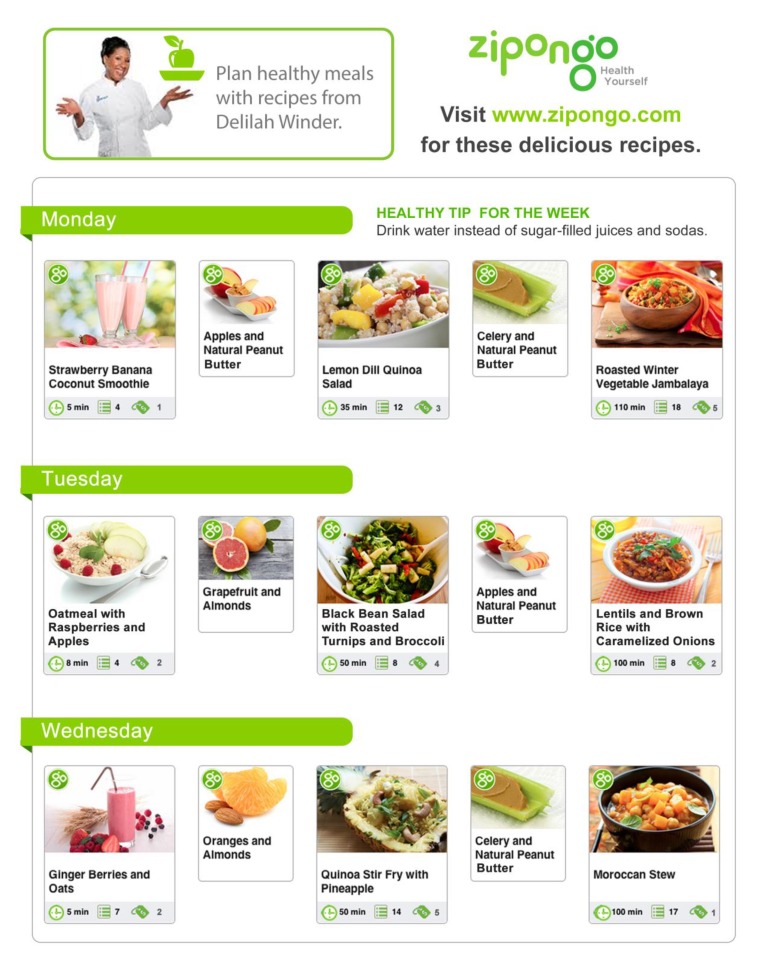
Sample Recommended Meal Plan Provided to Participants (ONLINE MATERIAL).

During the intervention, participants attended a two-hour session each week that focused on an overview of the 40-Day Daniel Fast, fitness and exercise, how stress can trigger chronic disease, including diabetes, heart health and stroke prevention, why taking your medication matters, and sharing program experiences. They also attended live cooking and food sampling demonstrations. Meal plans and corresponding recipes for each meal item were provided by the partners (Figure 1). The chef aligned the recommendations for the Fast to create culturally-relevant recipes for African Americans. A weekly grocery list linked to in-store discounts and loyalty card coupons was also provided. 

Daily or bi-weekly texts were sent with healthy eating tips and users could opt-in for additional daily recipe suggestions via text. Zipongo’s registered dietitian called participants to discuss personal nutrition and to ensure intervention materials were able to be utilized. At the conclusion of 6 weeks, participants continued to receive healthy eating and recipe tips via text from Zipongo.

### 2.2. Measures

Baseline, week 6 and 6-month follow-up measures were collected by the Drexel research team, Healthy Measures and Zipongo personnel. The primary study outcomes were changes in biomarkers (i.e., weight, LDL, HDL, waist circumference, body mass index (BMI), glucose, HgA1C). Body weight was collected using a calibrated scale with participants wearing no shoes and light clothes. Pre- and post-Fast measurements of cholesterol, glucose, waist circumference, and body mass index were also collected. HgA1c was assessed with a Unistik lancet from a fingerstick capillary whole-blood sample, collecting 40 microliters of blood using a capillary tube and measured using the A1cNow+. Total cholesterol was also measured from capillary whole blood sample. Blood pressure was assessed with an Omron BP760 automatic BP cuff, while participants were seated, relaxed for three minutes. 

Paper-pencil measures were administered and participants completed Sallis’ scale to measure social support from family and friends for diet and exercise behaviors [21]. Higher scores indicated more social support was provided by either a family or friend. They also completed the validated Food Choice Questionnaire (FCQ) [22,23]. 

The FCQ is designed to capture the multidimensional nature of food choice. Participants reported factors they considered important when selecting food items. The nine subscales within the FCQ are health, mood, sensory appeal, price, natural content, convenience, familiarity, ethical concern, and weight control. 

Overall, physical and mental health was assessed using the SF-12 Health Survey. The SF-12 assessed eight dimensions of physical and mental health outcomes: Physical functioning, Role Physical, Bodily Pain, General Health, Social Functioning, Role Emotional, Mental Health, and Vitality. A program assessment survey was used to collect participants’ feedback on the cooking demonstrations, food samples, web-based grocery deals, fitness information, disease prevention, medication matters, social support, and recipes. The participants used a 5-point Likert Scale (from strongly disagree to strongly agree) to report whether the material was relevant, easy to implement, easy to follow, and if the information helped to change their behaviors. 

Finally, self-reported data were collected on whether participants used the digital platform, how they used it, and whether they found it helpful during the 40-Day Journey. User engagement data was also gathered through telephone calls by a dietitian, text messages, and post-intervention paper surveys. 

### 2.3. Statistical Analyses

Respondent data from both churches were combined, a priori, to maximize sample size. Generalized linear models were run to test for statistical heterogeneity to validate the assumption of no difference between churches. For study measures where this assumption was not held, analysis was limited to the respective church. All data were input into SPSS version 20 and analyzed in SAS version 9.3 and STATA version 11.

Chi-square tests and simple means were used to compare descriptive data. Psychosocial and biometric measurements at baseline and the change in these measures at the end of study were examined for all participants with completed pre- and post-Fast measurements. The within-individual change from baseline was evaluated by using paired t tests for data distributed normally and matched-pair Wilcoxon signed rank sum tests for non-normally distributed data. 

## 3. Results

### 3.1. Participant Demographics

Across both churches, 135 participants were initially enrolled from Church 1 (*n* = 47) and Church 2 (*n* = 88). At the six-week culmination of the “40-Day Journey”, 69 participants completed surveys from Church 1 (*n* = 27) and Church 2 (*n* = 42). A majority of the participants were women (*n* = 57). The mean age across churches was 49.2 years (SD = 12.65 years). Participants from Church 1 were older (*m* = 51.1 years, SD = 11.96 years) than participants from Church 2 (*m* = 45.8 years, SD = 13.31 years). This difference in age was statistically significant (t(115) = 2.20, *p* = 0.03).

### 3.2. Biometric Data

The key outcomes of interest in this study were changes in biomarkers that indicate improvement in health. Table 1 presents these data. Participants experienced a statistically significant weight loss during the 40-Day Journey. They lost an average of 3.9 lbs (t(60) = 2.93, *p* = 0.0004). The decrease in waist circumference was 0.7 inches between pre (*m* = 41.50 in, SD = 6.69 in) and post assessment (*m* = 40.80 in, SD = 6.38 in). Overall, there was no statistically significant change in body mass index (BMI) between pre- and post-Fast. The mean Total Cholesterol (TC) dropped significantly between pre (*m* = 171.70 mg/dL, SD = 12.02 mg/dL) and post testing (*m* = 158.80 mg/dL, SD = 30.60 mg/dL). A Wilcoxon Signed Rank Test for non-normally distributed data indicated a statistically significant difference between the pre and post TC (t(62) = 5.17, *p* = 0.0001).

Zipongo text messaging continued after the completion of the 40-Day Journey (i.e., week six) through six months. Self-report data collected via survey also indicated that participants maintained improvements in mood and weight loss over six months. Overall, 90% of participants that completed the intervention stated that the program made them feel happier (*n* = 40), and 71% of participants reported losing weight, with an average weight loss of nine pounds (*n* = 35).

### 3.3. Blood Pressure Data by Church

While overall blood pressure did not approach significance for participants (Table 1), systolic blood pressure decreased by 9 mm/Hg between pre (*m* = 142 mm/Hg, SD = 19.9 mm/Hg) and post assessment (*m* = 133 mm/Hg, SD = 20.8 mm/Hg) amongst participants at Church 1. This change was statistically significant (t(22) = 2.46, *p* = 0.02). The decrease in pre (*m* = 88 mm/Hg, SD = 10.30 mm/Hg) and post (*m* = 83.90 mm/Hg, SD = 11.40 mm/Hg) of diastolic blood pressure amongst Church 1 participants approached significance (t(22) = 1.75, *p* = 0.09). 

### 3.4. SF-12 across Church and by Church

Out of the eight scales in the SF-12, three scales were statistically significant across (Table 2) both churches between pre- and post-Fast assessments. Participants reported statistically improved mean scores in social functioning between pre (*m* = 72.75, SD = 28.45) and post (*m* = 80.65, SD = 30.08; t(23) = 3.00, *p* = 0.01). Mental health scores also improved between pre (*m* = 69.41, SD = 22.94) and post (*m* = 75.81, SD = 23.49); t(23) = 2.65, *p* = 0.01). Additionally, vitality scores improved significantly from pre (*m* = 51.57, SD = 25.55) to post (*m* = 62.58, SD = 25.69; t(23) = 2.33, *p* = 0.02). The breakdown by church show that the statistically significant improvements in mental health (*p* < 0.01) and vitality (*p* < 0.01) were for Church 2 participants and the improvements in social functioning were for Church 1 participants (*p* < 0.05).

### 3.5. Food Choice

Subscales that emerged as statistically significant from pre- and post-Fast assessments were natural content, convenience, familiarity, and weight control. Participants reported natural content was an important factor in their food choice at post (*m* = 3.32, SD = 0.62) compared to pre assessment (*m* = 3.04, SD = 0.75; (t(59) = 3.12, *p* = 0.003; Table 3). Also, between pre (*m* = 3.30, SD = 0.68) and post (*m* = 3.17, SD = 0.63), participants reported that convenience became a less important factor in food choice (t(59) = 2.18, *p* = 0.03). Similarly, participants began to eat less familiar foods which indicated that they were trying new foods and not eating their usual foods between pre (*m* = 2.66, SD = 0.91) and post (*m* = 2.44, SD = 0.95; (t(59) = −2.62, *p* = 0.01). Finally, weight control was statistically significant between pre (*m* = 3.25, SD = 0.83) and post (*m* = 3.47), t(58) = 2.02, *p* = 0.048). These all represent positive changes with respect to knowledge and food choice behaviors directly related to participating in the 40-Day Journey. 

### 3.6. Zipongo: Digital Engagement for Healthier Eating 

Initially, 181 participants consented and enrolled in the Zipongo platform; 57 participants (31%) opted to receive emails, and average open rate was 30%. Additionally, 155 participants (86%) opted to receive text messages. Of these, 66% opted to receive daily text messages and 34% opted to receive bi-weekly text messages. Also, 57% and 19% of those receiving daily and bi-weekly tips, respectively, texted back. Some text participants (*n* = 48; 31%) also opted to receive additional daily recipe texts. These participants were even more responsive, with 100% texting back at least once throughout the 6 weeks of recipes. These responses showed their interest in and attitudes towards the health tips and recipes and that they were changing eating behaviors and following suggestions from the tips (Table 4).

At the conclusion of the text program, participants were also asked to rate their satisfaction with Zipongo through text message and through a follow-up survey during biometric screening. From the written assessments, 72% of participants reported that Zipongo’s web-based grocery deals and recipes increased their knowledge about healthy nutrition, and 66% said that these deals helped change eating behaviors. Further when prompted to respond on a 5-point Likert through text message on, “How helpful have our tips have been?”, 98% responded with 4 or 5, indicating high satisfaction (*n* = 49). On a 10-point scale, participants gave an average rating of 9 for whether they would recommend Zipongo to a friend (*n* = 39). Though assessed on different scales, the combined quantitative and qualitative engagement and satisfaction data suggest that SMS messaging had a greater impact than web and email, for this population. 

## 4. Discussion

### 4.1. Program Assessment

Overall, participants rated the program highly across all domains. Individuals agreed that the information presented from Cooking Demonstrations and Food Samples were relevant (93%, 96.5%, respectively). It is of note that the food samples provided seemed to be an important component for participants as they were able to taste-test novel foods. This demonstrated how slight modifications for traditional foods could make a potentially positive impact on food choices. Ninety-four percent of participants surveyed stated that this could have an influence as they consider changing their eating behaviors. Finally, the fitness, disease prevention, and medication topics covered were also highly rated as being relevant, easy to follow and influential in increasing knowledge. 

This study demonstrates the importance of integrating multiple approaches to improve health, particularly in urban settings. In order to begin to address reducing health disparities, underserved and underrepresented minority groups may require hybrid approaches, such as this project as it fits within community culture, context and religious practices and religious norms. The core interventions of the 40-Day Journey included in-person classes, personalized digital recipe and grocery list recommendations, and biometric data collection in a familiar setting. Each of these components may be critical in this model to engage a hard to reach population at high risk for many chronic conditions. By working with individuals in the context of a trusted setting (i.e., church) we were able to bring traditional health education together with technology. In addition, cooking demonstrations by the chef paired with personalized guidance for specific foods to buy, cook and eat via image-based print materials for web, email and text messaging were critical to the program success. The call from the dietitian helped participants navigate and understand the recipes and meal plans. Research has shown that only prescribing a diet and offering nutrition tracking, or health education alone but lacking a maintenance program can impact the success of adoption of new health knowledge and healthy behaviors [24,25,26].

### 4.2. Limitations

This study recruited church members from churches who partnered with the AmeriHealth program. Thus, this was a convenience sample of church members which may limit generalizability to a broader non-church going population. The overall sample included more middle aged women than men which may also limit our understanding of younger populations as well as men in urban settings. Most church-based interventions in African American communities have similar demographics for church attenders with women more likely to regularly attend. Finally, while there was support for individuals in using the nutrition-based application on their phone, individuals who may have had difficulty with technology may not have used all features available to them in the app.

## 5. Conclusions 

To our knowledge, this is the first community research evaluation of the Daniel Fast in conjunction with a digital engagement component in a church-based setting. The 40-Day Journey results are promising given the positive impact on blood pressure, cholesterol, weight outcomes and mental wellness outcomes. This project also demonstrates how multiple partners can come together (university-community-industry) for evaluation projects in the community. 

The authors believe that there may be interest in these types of projects to introduce and initiate healthful eating as it is connected to faith beliefs. Understanding the types of programs used in faith settings with an urban African American population may be of value, particularly as the DanielFast continues to gain momentum with churches across the country. Understanding more about the incentives to engage in a healthy lifestyle change in a familiar setting with the support of others in their church community adds to the research literature on strategies to reduce health disparities. Finally, adding a technology component with high touch points between sessions may also be cost effective and nationally scalable across diverse communities. Further studies are needed to evaluate longer-term follow-up at 12 and 18 months, and to understand the relative contributions of different elements of the 40-Day Journey integrated intervention for participants

## Figures and Tables

**Table 1:** Participants’ Biometric Data.

	Pre-Test Mean (SD)	Post-Test Mean (SD)	df	T-Score	*p*-Value
Weight (lbs)	210.00 (52.00)	206.10 (51.96)	60	2.93	0.005 *
Waist circumference (in)	41.50 (6.69)	40.80 (6.38)	61	1.83	0.07
BMI (kg/m^2^)	34.84 (7.32)	34.52 (7.56)	61	1.35	0.18
Total Cholesterol (mg/dL)	171.7 (12.02)	158.8 (30.60)	62	5.17	0.0001 *
Glucose (mg/dL)	126.95 (51.30)	117.60 (35.88)	61	1.81	0.071
Systolic BP (mm/Hg)	134.59 (19.16)	130.92 (19.05)	62	1.64	0.11
Diastolic BP (mm/Hg)	83.03 (10.56)	82.76 (10.51)	62	0.18	0.86

* *p* = 0.03 (Wilcoxon Signed-Rank test).

**Table 2:** SF-12 Results for Congregants.

SF-12 Domain	Pre-Test Mean (SD)	Post-Test Mean (SD)	df	T-Score	*p-*Value
Physical functioning	61.46 (37.58)	67.71 (36.47)	23	0.97	0.34
Role physical	72.92 (44.18)	77.08 (38.95)	23	0.46	0.65
Bodily pain	73.95 (30.82)	78.13 (28.85)	23	0.7	0.49
General health	48.95 (20.16)	46.88 (16.99)	23	−0.49	1
Social functioning	69.79 (33.77)	83.33 (27.25)	23	3	0.01 *
Role emotional	68.75 (43.77)	77.08 (36.05)	23	1.16	0.26
Mental health	70.00 (22.26)	79.17 (21.45)	23	2.65	0.01 *
Vitality	52.50 (24.18)	65.00 (23.77)	23	2.33	0.03 *

* *p* < 0.05.

**Table 3:** Food Choice Questionnaire (FCQ) Results for Participants.

FCQ Dimension	Pre-Test Mean (SD)	Post-Test Mean (SD)	df	T-Score	*p*-Value
Natural content	3.04 (0.75)	3.32 (0.62)	59	3.12	0.003 *
Convenience	3.30 (0.68)	3.17 (0.63)	59	2.18	0.03 *
Familiarity	2.66 (0.91)	2.44 (0.95)	59	−2.62	0.01 *
Weight control	3.25 (0.83)	3.47 (0.57)	58	2.02	0.048 *
Ethical concern	2.51 (1.05)	2.60 (0.91)	59	0.8	0.43
Price	3.24 (0.74)	3.13 (0.76)	59	−1.3	0.2
Mood	2.95 (0.90)	2.83 (0.95)	59	1.15	0.26
Sensory appeal	3.30 (0.67)	3.26 (0.68)	59	−0.061	0.54
Health	3.27 (0.73)	3.40 (0.62)	59	−1.52	0.13

* *p* < 0.05.

**Table 4:** Text Responses from 40-Day Journey Participants.

**Healthy Tips and Recipe Responses**
“Good information for me to know. Thank you”
“I luv [*sic*] the daily tips pls [sic] cont [*sic*] thank you”
“Soup was delicious, great using veg [*sic*] stock”
“I am enjoying the recipes. They are easy and quick to make. I have a new appreciation for beans. Please keep them coming. I like new dishes. Thanks!”
**Responses Indicating Behavior Change**
“I made some soup and I ate lots of salad last week. No meat!!!”
“I made ur [*sic*] wonderful meals. My favorite so far is the black bean chili and lentil/sweet potato”
“eating more healthier, made spagetti [*sic*] squash and califlower [*sic*]with parsnips blended together instead of mash potatoes topped with veggie chee[se]” [*sic*]
“Thank u [*sic*] for the Healthy meals!!!!! I m [*sic*] on day 3 eating veggies Just loving it. Thank u [*sic*]again”
